# Structural and biophysical comparisons of the pomalidomide- and CC-220-induced interactions of SALL4 with cereblon

**DOI:** 10.1038/s41598-023-48606-3

**Published:** 2023-12-12

**Authors:** Xiaolei Ma, Barbara Leon, Elizabeth Ornelas, Dustin Dovala, Laura Tandeske, Catherine Luu, Gwynn Pardee, Stephania Widger, Jonathan M. Solomon, Rohan E. J. Beckwith, Heinz Moser, Matthew C. Clifton, Charles A. Wartchow

**Affiliations:** 1 Global Discovery Chemistry, Novartis Biomedical Research, Emeryville, CA 94608 USA; 2 Discovery Sciences, Novartis Biomedical Research, Emeryville, CA 94608 USA; 3 Discovery Sciences, Novartis Biomedical Research, Cambridge, MA 02139 USA

**Keywords:** Molecular biophysics, X-ray crystallography, Biochemistry, Biochemistry

## Abstract

The design of cereblon-binding molecular glues (MGs) that selectively recruit a desired protein while excluding teratogenic SALL4 is an area of significant interest when designing therapeutic agents. Previous studies show that SALL4 is degraded in the presence of IKZF1 degraders pomalidomide, and to a lesser extent by CC-220. To expand our understanding of the molecular basis for the interaction of SALL4 with cereblon, we performed biophysical and structural studies demonstrating that SALL4 zinc finger domains one and two (ZF1-2) interact with cereblon (CRBN) in a unique manner. ZF1 interacts with the *N-*terminal domain of cereblon and ZF2 binds as expected in the *C-*terminal IMiD-binding domain. Both ZF1 and ZF2 contribute to the potency of the interaction of ZF1-2 with CRBN:MG complexes and the affinities of SALL4 ZF1-2 for the cereblon:CC-220 complex are less potent than for the corresponding pomalidomide complex. Structural analysis provides a rationale for understanding the reduced affinity of SALL4 for cereblon in the presence of CC-220, which engages both ZF1 and ZF2. These studies further our understanding of the molecular glue-mediated interactions of zinc finger-based proteins with cereblon and may provide structural tools for the prospective design of compounds with reduced binding and degradation of SALL4.

## Introduction

The design of highly selective low-molecular weight glues (MGs) that cause the degradation of a specific protein and exclude others is an area of significant interest in the drug discovery process. Both globular proteins and C2H2 zinc-finger based transcription factors that contain a beta-turn are known to form complexes in the presence of MGs known as immunomodulatory imide drugs (IMiDs) that bind to CRBN, leading to ubiquitination and proteosome-mediated degradation^1–4^. MGs can recruit or degrade more than one target; for example, pomalidomide (POM) and CC-220 recruit and degrade transcription factor IKZF1 along with SALL4 and several other targets^3,5,6^. The origin of selectivity is an ongoing area of interest since off-target effects can lead to undesirable consequences or challenges in understanding the biology associated with each additional target that is degraded.

With respect to the molecular glue-induced interaction of zinc finger transcription factors with cereblon, structural studies show that MGs bind to the *C-*terminal domain of cereblon and induce conformational changes that result in a new surface that interacts with zinc fingers containing a beta turn motif with sequence CXXCG^7–9^. Structural studies of the MG-induced binding to cereblon include single ZF domains from IKZF1^3,7^, IKZF2^9,10^, SALL4^8,11^, and ZNF692^3^. Biochemical studies with IKZF1 in the presence of pomalidomide indicate that ZF2-3 binds more potently than ZF2 alone^3^, suggesting that for these proteins, more than one zinc finger contributes to interactions with the CRBN:MG binary complex. Likewise, for IKZF2 ZF2-3, binding to cereblon is induced by DKY709, and structural and SPR binding studies reveal that this interaction also involves two zinc fingers^10^. In this example, both zinc fingers interact with the *C-*terminal domain and several ZF3 residues likely form additional interactions with cereblon that contribute to the observed improvement in potency relative to ZF2 alone.

SALL4 degradation is linked to developmental abnormalities and avoiding off-target degradation is desireable^12^. Notably, CC-220, a potent degrader of IKZF1, causes less degradation of SALL4 than pomalidomide^5^. Like ZF2-3 of IKZF1 and IKZF2, SALL4 ZF1-2 binds more tightly to CRBN:MG complexes than ZF2 alone^6^. Furthermore, the addition of *C-*terminal residues PQVKA to SALL4 ZF2 results in increased degradation relative to ZF2 alone^13^. The binding mode of SALL4 ZF1-2 to CRBN:MG complexes must be distinct from the IKZF family members since the location of ZF1 is at the *N-*terminus instead of the *C-*terminus of the primary zinc finger.

To further understand the reported selectivity of pomalidomide and CC-220 and the interactions between the SALL4 zinc finger domains and CRBN:MG complexes, we performed biophysical and structural studies with SALL4 zinc finger domains. To understand the selectivity of pomalidomide and CC-220 as well as the contributions of ZF1 and ZF2 to the binding of ZF1-2, we determined the binding affinities of single and double zinc finger domains to CRBN:MG complexes by SPR. To understand the structural elements that confer the binding affinities and selectivity observed in these studies, we determined the structures of CRBN complexes with pomalidomide or CC-220 and SALL4 ZF1-2 domains. Comparisons of structural features provide molecular insights into the interactions and affinities of SALL4 zinc fingers with these CRBN:MG complexes.

## Results

### Determination of the MG-mediated binding affinities of SALL4 zinc finger domains to cereblon by SPR

**Figure 1:**
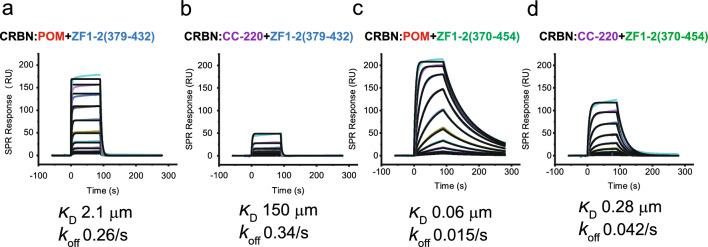
Representative SPR binding data with 1:1 kinetic modelling for the interaction of SALL4 ZF1-2 domains with DDB1:CRBN complexed with pomalidomide (POM) or CC-220. Each plot shows a 2X dilution series of ZF1-2(379–432) or ZF1-2(370–454) at top concentrations and 25 μM and 1 μM, respectively. (**a**), (**b**) interaction of SALL4 ZF1-2(379–432) with the CRBN:POM and CRBN:CC-220 complexes, respectively. (**c**), (**d**) interaction of SALL4 ZF1-2(370–454) with the CRBN:POM and CRBN:CC220 complexes, respectively.

To identify the minimal SALL4 binding domain that interacts with human cereblon complexed with IMiD molecules pomalidomide and CC-220, we examined the SPR binding affinities of SALL4 zinc finger domains 1, 2, and ZF1-2 to DDB1:CRBN:MG complexes (Table 1 and Fig. 1). In these experiments, the concentration of pomalidomide or CC-220 was fixed at 5 μM, which results in near saturation of cereblon since the *K*_D_ values for the interaction of these molecules with DDB1:CRBN are 170 ± 0.30 and 9 ± 0.9 nM, respectively (Fig. S1). Measuring the interaction of a dilution series of ZF(s) with the DDB1:CRBN:MG complex results in the determination of the affinity of the ZF(s) for the DDB1:CRBN:MG complex. For the DDB1:CRBN:POM complex, ZF1(370–409) shows no detectable binding at 25 μM, and ZF2(405–432) binds weakly to the CRBN:POM complex with a *K*_D_ of 67 μM. Extension of ZF2 to include the *C-*terminal sequence PQVKA identified previously in cellular degradation studies^13^ resulted in a *K*_D_ of 0.98 μM, a 68-fold improvement in affinity of ZF2(405–437) relative to ZF2(405–432). Further extension of the *C-*terminus to ZF2(405–454) did not result in further improvements. The addition of ZF1 to ZF2(405–432) and ZF2(405–454) results in 21- and 11-fold improvements in affinity for the interaction of these ZFs with the CRBN:POM complex, respectively, suggesting that both ZF1 and ZF2 interact with cereblon. The affinity of ZF1-2(370–454) is 52-fold more potent than ZF1-2(379–432), likely due to the inclusion of the PQVKA residues that resulted in more potent affinity for ZF2(405–437). Analysis of the same ZFs with the DDB1:CRBN:CC-220 complex resulted in the same trends, but the affinities were less potent than for the interaction of ZF2 alone and ZF1-2 with the CRBN:POM complex. Overall, these findings are consistent with engagement of both SALL4 ZF1 and ZF2 with CRBN:MG complexes as well as the previously reported involvement of *C-*terminal ZF2 extension PQVKA. Since the affinity of ZF2 alone is more potent than ZF1, ZF2 is the primary determinant of binding affinity in the ZF1-2 complex. Furthermore, the differences in affinities of SALL4 ZF domains for the DDB1:CRBN:CC-220 complex are consistently weaker than those in the corresponding DDB1:CRBN:POM complex suggesting that the interactions of these ZF domains with the CRBN:MG complex are distinct.

**Table 1:**
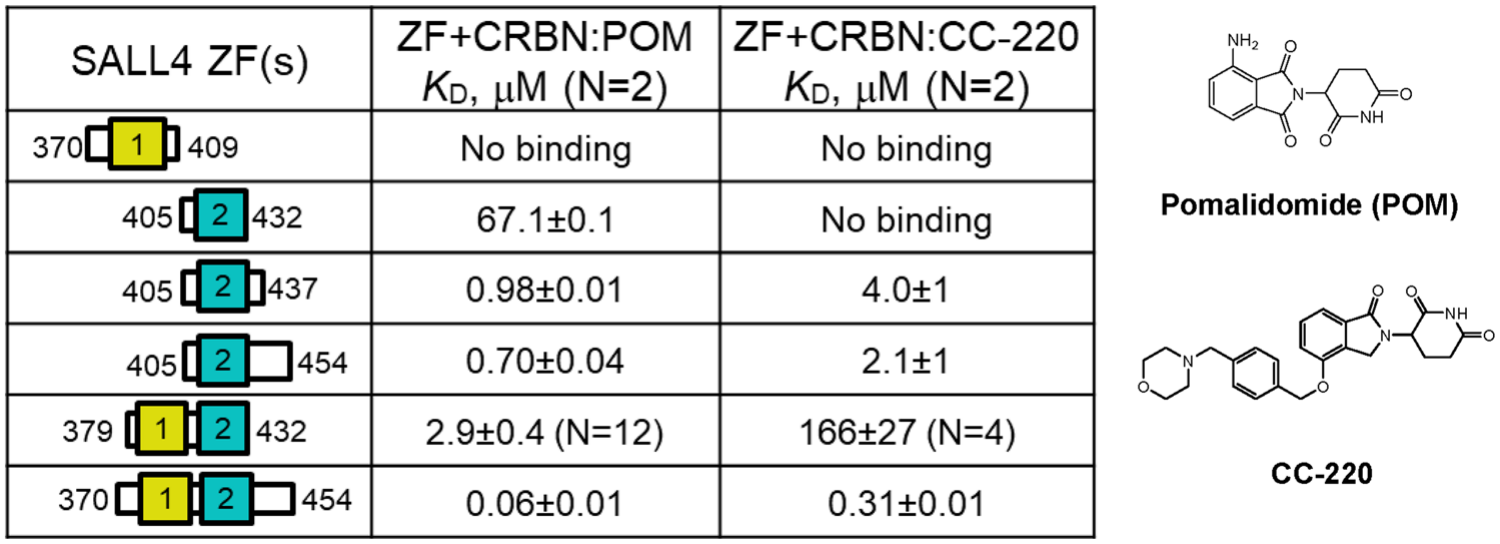
SPR affinities for the interaction of SALL4 zinc fingers 1, 2, or 1–2 with immobilized DDB1:CRBN complexed with pomalidomide (POM) or CC-220 at 5 μM.

“No binding” indicates no response for ZF1(370–409) and ZF2(405–432) at 25 μM. All results are duplicates (N = 2) unless noted.

### SALL4 ZF1 and ZF2 interact with CRBN:MG complexes

**Figure 2:**
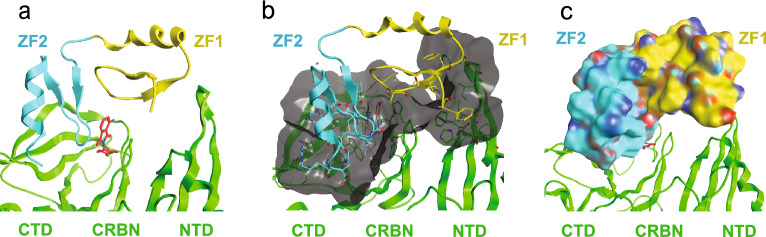
SALL4 ZF1-2 interacts with CRBN:POM in the DDB1:CRBN:POM:ZF1-2(379–432) complex. (**a**) Ribbon diagram showing the interaction of SALL4 ZF2 (cyan) and ZF1 (yellow) with the *C-*terminal IMiD-binding and *N-*terminal domains of cereblon, respectively, in the presence of pomalidomide (red). (**b**) The cereblon contact surface area (gray) and SALL4 residues that are proximal with cereblon (**c**) Space-filling model of SALL4 ZF1-2 in the CRBN:POM:ZF1-2(379–432) complex.

To understand the interactions of SALL4 ZF1 and ZF2 with CRBN suggested by SPR experiments, X-ray structures of ZF1-2(379–432) in a quaternary complex with DDB1:CRBN and CC-220 or pomalidomide were determined at 2.9 Å and 3.0 Å resolution, respectively (PDB entries 8U15 and 8U16). To understand the impact of domain extensions, including the *C-*terminal PQVKA sequence, the X-ray structure of ZF1-2(370–454) with the DDB1:CRBN:POM complex was determined at 3.1 Å (PDB entry 8U17). Diffraction data and refinement statistics for these structures are in Table S1. The complexes show the expected interactions of ZF2, which contains the known CXXCG recognition motif, with the *C*-terminal IMiD-binding domain (CTD) of CRBN. In addition, interactions occur between ZF1 and the *N-*terminal domain (NTD) of CRBN in the “closed” form of CRBN, where the NTD and the CTD are proximal. The contact areas between SALL4 ZF1 and ZF2 and CRBN in the CRBN:POM complex are 225 Å^2^ (38%) and 367 Å^2^ (62%), respectively. General cereblon contacts with ZF2 involve several strands of beta-sheet and the IMiD-interacting loop defined by cereblon N351-Y355. The cereblon contacts with ZF1 are more limited, involving one side of a beta hairpin (F150-I154) and residues near a helical region, including F102. Additionally, ZF1 and ZF2 share intramolecular contacts between these domains. These general structural features are exemplified for the DDB1:CRBN:POM:ZF1-2(379–432) complex in Fig. 2.

Key interactions of ZF1-2(379–432) with the CRBN:POM complex include ZF2 β-turn residues 411–417, which make numerous backbone and/or side chain interactions within ~ 4 Å of CRBN or POM. CRBN residues that interact with ZF2 include N351, H357, W400, P352, H353, Y355, and W386 and these residues were identified and validated by mutagenesis in a previous study with ZF2 alone^8^. SALL4 ZF2 helix residues H428, F429, R431 and H432 interact with the CRBN CTD. ZF1 residues Y380, C387, K389, V390, F391 and G392 interact with the CRBN NTD, and CRBN residues F102, P104, I152, F150, G151, and H353 residues are in proximity to SALL4 ZF1. ZF1 and ZF2 also contain intramolecular contacts between these two domains, including ZF1 residues K385 and Y386, which form contacts with ZF2 residues P409, F410, and V411. Contacts between pomalidomide and ZF1 are not observed.

The structure of DDB1:CRBN:POM:ZF1-2(370–454) is similar to the corresponding POM/ZF1-2(379–432) structure described above; RMSDs for CRBN-based structural alignments are 0.6 and 0.9 Å for CRBN and ZF1-2, respectively (Fig. S2). For ZF1-2(370–454), *C*-terminal residues P433 and portions of Q434 are visible in the X-ray structure, and P433 interacts with CRBN S420 (Fig. S3). A model of the interaction of ZF2 residues P433 and Q434 suggests additional contacts with CRBN residues M88, S420, V128, Q129, and Y355 and these interactions may be responsible for the increased potency observed for ZF2(405–437) and ZF1-2(370–454) relative to ZF1-2(379–432) and ZF2(405–432), which lack these residues.

### CC-220 engages ZF1 and ZF2 in the CRBN:CC-220:SALL4 ZF1-2(379–432) complex

**Figure 3:**
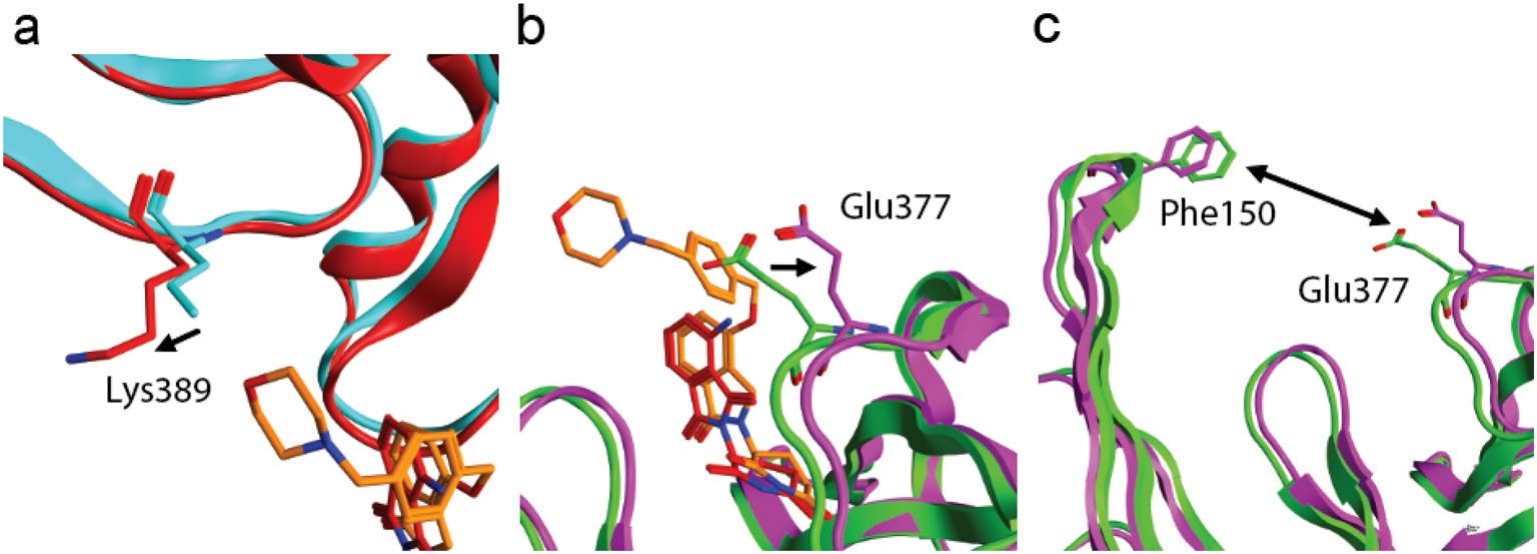
Comparison of key features in CRBN:MG:ZF1-2 complexes. (**a**) Representation of the location of ZF1 K389 atoms visible in structures for complexes CRBN:CC-220:ZF1-2(379–432) (magenta/orange/red) and CRBN:POM:ZF1-2(379–432) (green/red/cyan). Cereblon is not visible. (**b**) The locations of E377 in these complexes. ZF1-2 is omitted for clarity. (**c**) Comparison of the differences in locations of the NTD (left) and CTD (right) using corresponding residues F150 and E377, respectively. ZF1-2, pomalidomide, and CC-220 are omitted for clarity.

In comparing the CC-220-based structure to the corresponding pomalidomide structure, significant structural similarity for both cereblon and ZF1-2 in the DDB1:CRBN:CC-220:ZF1-2(379–432) complex is observed, with RMSD of 0.9 and 1.1 Å for cereblon and ZF1-2, respectively, for the superposition of these proteins (Fig. S2). Notable differences in these two structures include the location of ZF1 K389 and cereblon residue E377, which shift in the presence of CC-220. With respect to K389, the distal morpholine ring of CC-220 engages the side chain of K389, which is not engaged by pomalidomide because it lacks the phenyl ring and morpholine ring that are present in CC-220. The shift in the position of visible K389 Cδ in the CC-220 structure is 1.7 Å away from the IMiD-binding pocket toward F150 (Fig. 3A). The X-ray density for CC-220 and the adjacent side chain of K389 are well-resolved, and the distance of the morpholine oxygen atom to Cδ of K389 is 3.4 Å. The comparable distance for the well resolved Cδ in the superposed pomalidomide X-ray structure is 2.6 Å, which suggests that the different location of the side chain atoms of K389 in the CC-220 structure is a result of a steric clash with CC-220 and the side chain of K389. Neither Cε nor the zeta amino group of K389 are visible in the X-ray structure with smaller pomalidomide, suggesting that these atoms adopt multiple conformations in the absence of larger CC-220. To assess the relevance of these key structural observations with K389, we examined the interaction of a K389A mutant of SALL4, ZF1-2(379–432) K389A, with CRBN:MG complexes. The affinity of the K389A mutant for the CRBN:POM complex is similar to native ZF1-2(379–432); *K*_D_ values are 4.4 ± 0.3 (N = 6) and 2.9 ± 0.4 (N = 12) μM, respectively. These results are consistent with the observed lack of interaction of pomalidomide with K389 in the structure. For the corresponding CRBN:CC-220 complex, however the affinities of ZF1-2(379–432) K389A and native ZF1-2(379–432) were 64 ± 3.7 (N = 6) and 166 ± 27 (N = 4) μM, respectively. This 2.6-fold difference is consistent with the direct interaction of CC-220 with ZF1 K389 and the improvement in affinity for the K389 mutant is consistent with removal of a steric clash with CC-220, which is likely lacking in the K389A mutant.

The other significant difference in these two structures occurs at cereblon residue E377, which engages the distal amino group of the phthalimide ring of pomalidomide. In the CC-220 structure, this residue shifts away from the glue in the presence of CC-220 due to its phenyl ring, which clashes with E377, based on comparison to the location of E377 in the corresponding pomalidomide structure. Engagement of E377 by CC-220 results in a 1.5 Å shift in Cβ away from the glue (Fig. 3B). E377 is part of a beta hairpin that contains W380, which is part of the cereblon IMiD binding pocket; thus, the shift of E377 results in a shift in the location of the glue in the pocket which is expected to impact the interaction of SALL4 ZF2 with the cereblon:CC-220 surface relative to the corresponding interaction with the CRBN:POM surface. Overall, these changes, and likely others, result in a change in the proximity of the *C-*terminal and the *N-*terminal domains of cereblon by ~ 1.8 Å, measured using Cβ of both E377 and F150, which is proximal to ZF1, respectively (Fig. 3C). These changes in structure likely impact the interactions of ZF1-2 with cereblon, reducing the affinity of the interaction with CRBN:CC-220 complexes relative to the corresponding POM complex.

## Discussion

The discovery that the IMiD class of molecular glues that bind to cereblon and cause selective recruitment and degradation of zinc-finger based transcription factors such as IKZF1 and others enables new opportunities for this target class, which has been historically challenging due a lack of tertiary protein structure and ligandable pockets for these largely disordered proteins^3^. Studies have shown that protein degradation is chemotype dependent and that single atom changes among lenalidomide and pomalidomide result in the degradation of different protein populations^3,6^, yet the origin of the observed selectivity for a given target (or targets) is unclear. The ability to design cereblon ligands that selectively degrade a desired target with minimal to no degradation of other proteins is important for understanding the biology of the target of interest and minimizing undesired biological consequences due to off-target degradation.

Accordingly, we sought to understand the interaction of SALL4 with CRBN in the presence of molecular glues pomalidomide and CC-220. To determine the minimal binding domain recruited by these molecules, we chose to focus on ZF2, which is essential for degradation, and adjacent ZF1, which was implicated in biochemical binding studies^6,8,12^. Through measurement of the binding affinities of SALL4 ZF constructs for CRBN:MG complexes, we confirmed the improvements in potency suggested in biochemical assays for ZF1 and for a *C-*terminal extension that includes residues PQVKA implicated in a previous study^13^. Structures of these complexes reveal that ZF1 interacts with the CRBN NTD, and ZF1-2 containing *C-*terminal PQ residues is likely the minimal binding domain of SALL4. This is the first structural example where an *N*-terminal ZF that is adjacent to the primary CXXCG-containing ZF contributes to binding in these complexes. Additionally, it is the third binding mode for ZFs to CRBN:MG complexes adding to our understanding of the binding of single zinc fingers such as IKZF1^3,7^ and ZNF692^3^, and the binding of two zinc fingers including *C-*terminal ancillary ZF3 in the IKZF2 ZF2-3 complex^9,10^.

With respect to understanding the interactions of ZF1-2 with CRBN:MG complexes, the present studies provide new insights into the role of multi-zinc finger domains in CRBN:MG:SALL4 complexes as well as a rationale for the selectivity of two distinct chemotypes, pomalidomide and CC-220. Since the binding affinity of ZF2(405–437) is relatively potent (*K*_D_ 0.98 μM), and ZF1 binding is not detectable at 25 μM (*K*_D_ estimated at > 50–100 μM), ZF2 interactions contribute to most of the binding affinity in ZF1-2. ZF1 interactions contribute ~ tenfold improvement in affinity for the interaction of ZF1-2 with the CRBN:POM complex. The *C-*terminal residues P433 and Q434 likely contribute significantly to this interaction; ZF2(405–437) is 68-fold more potent than ZF2(405–432) alone, and these residues are visible in the CRBN:POM:ZF1-2(370–454) structure.

With respect to selectivity, the weaker potencies observed for the interactions of SALL4 ZFs in the CRBN:CC-220 complex are consistent with previous reports of weaker degradation induced by this molecule^5^. These weaker interactions are also consistent with notable interactions in CRBN:MG:ZF1-2 complexes observed in our structural studies, including the interaction of MGs with CRBN E377 and SALL4 ZF1 K389. In the CRBN:CC-220:ZF1-2(379–432) complex, CRBN E377 is displaced relative to its position in the corresponding POM structure by 1.5 Å at Cβ as a result of a clash with the phenyl ring of CC-220. Since the E377 carboxylate interacts with the amino group of POM and does not interact with ZF2 directly, the difference in selectivity is likely due to subtle changes in the backbone atoms attached to E377 in the presence of CC-220, which impact nearby CRBN:ZF2 contacts. The selectivity can be further explained by the interaction of CC-220 with SALL4 ZF1 residue K389, which results in a steric clash. These two interactions, and possibly others, result in a widening of the CRBN NTD and CTD by ~ 1.8 Å, which likely impacts other interactions of cereblon with SALL4 ZF1-2. In summary, the origins of selectivity of MGs pomalidomide and CC-220 for the recruitment of ZF1-2 include the interactions of MGs with cereblon residues in the *C-*terminal glue-binding domain, interactions of CC-220 with ancillary ZF1 and possibly changes in other interactions due the widening of the distance between the NTD and CTD of cereblon.

In conclusion, we define the structural context of a minimal binding domain of SALL4 with CRBN:MG complexes that includes ZF1-2. This interaction includes the primary zinc finger, ZF2, that binds at the CRBN:MG interface in the *C-*terminal domain and an ancillary zinc finger, ZF1, that binds to the CRBN *N-*terminal domain. ZF2 is the primary driver of affinity, and ancillary ZF1 contributes ~ tenfold improvement in the affinity of ZF1-2 to CRBN:POM complexes. The binding affinity of ZF1-2 is weaker for the CRBN:CC-220 complex relative to the CRBN:POM complex due to interactions of ZF2 with the CTD, and the morpholine ring in CC-220 likely causes unfavorable interactions with ancillary ZF1. These observations and the structures reported herein may be extended to the modeling and design of additional molecular glues for therapeutic indications where the degradation of SALL4 is undesirable. These results expand our understanding of the interaction of zinc-finger based transcription factors with cereblon in the presence of molecular glues.

## Methods

### Protein production and purification

Human CRBN constructs were prepared as previously described^11^. Cereblon (residues 68–442) tagged with *N*-terminal ZZ-His fusion^14^ were co-expressed with human DDB1ΔBPB in Sf21 cells (Expression Systems) with 100 μM zinc acetate supplemented ESF921 medium. ZZ is a protein commonly used to improve protein expression levels, and His refers to a His-6 tag for capture on nickel columns for protein purification. For SPR binding assays, full-length human CRBN constructs (residues 1–442) were generated with a non-cleavable *N*-terminal Avi-tag^15^ with a 6xGS linker in addition to the upstream solubility tag. Full-length CRBN was co-expressed with human DDB1 (residues 1–1140). Avi-tagged CRBN was labeled at 100% efficiency with biotin using BirA enzyme. Expression pellets were stored at − 80 °C until processed. Frozen cells were lysed by homogenization at pH 7.5. The soluble fraction was purified with histidine-affinity, ion-exchange, and size-exclusion chromatography. Protein was concentrated to ~ 25–30 mg/mL in 20 mM HEPES, pH 7.0, 250 mM NaCl, 2 mM TCEP.

Human SALL4 constructs (379–432, 370–409, 405–432, 405–454 and 405–437) were *N-*terminally tagged with Twin-Strep-ZZ^16^ in a pET vector and were expressed in *E. coli* HiControlBL21(DE3) cells (Biosearch Technologies cat# 60435-1) in TB medium supplemented with 100 μM zinc acetate. Twin-Strep is an affinity tag that binds to streptavidin. Frozen E. *coli* cells were lysed via sonication or microfluidizer at pH 8.0. SALL4 proteins were purified from the soluble fraction with Streptavidin-affinity. The affinity tag was cleaved by TEV protease and untagged protein was isolated via size-exclusion chromatography. Protein was concentrated to 0.5–4 mg/mL in 20 mM HEPES, pH 7.5, 150 mM NaCl, 1 mM TCEP. Human SALL4 ZF1-2(370–454) was expressed in Sf21 cells (Expression Systems) with ESF921 medium. Frozen Sf21 cells were lysed by homogenization at pH 8.0 and soluble protein was isolated by Streptavidin-affinity with on-column TEV cleavage overnight 4 °C. The protein was processed with HisTrap, in tandem with the Streptavidin-affinity column followed by further purification by size-exclusion chromatography. Human SALL4 ZF1-2G protein was concentrated to ~ 2 mg/mL in 20 mM HEPES, pH 7.5, 250 mM NaCl, %5 Glycerol, 1 mM TCEP. For reference, a representation of full length SALL4 and examples of the constructs used herein are shown in Fig. S4.

### SPR characterization of SALL4 interactions with CRBN:MG complexes

In a typical experiment, DDB1:CRBN (“CRBN”) is immobilized on the sensor surface on a Biacore 8k SPR instrument. For the interaction of pomalidomide or CC-220 with CRBN, a 2X dilution series was injected in serial starting with two blanks followed by the lowest concentration. For the interaction of SALL4 zinc finger domains with CRBN, the proteins were diluted in buffer containing 5 μM pomalidomide or CC-220 and were injected in serial to monitor the association and dissociation of these proteins to CRBN in the presence of 5 μM pomalidomide or CC-220. The *K*_D_ values for the interaction of MGs pomalidomide or CC-220 with CRBN were 170 and 9 nM respectively. These conditions result in measurement of the interaction of SALL4 ZF1-2 or another construct with CRBN:POM or CRBN:CC-220 complex. In detail, the method includes immobilization of DDB1:*N*-avi-biotin-CRBN on a streptavidin-coated Biacore sensor to ~ 5000–7000 resonance units (RU), and the surface was exposed to 1 µM biocytin to block unoccupied streptavidin sites prior to analyses. The binding of SALL4 zinc finger domains to CRBN:MG complexes was measured in PBS buffer at pH 7.2 containing 5% glycerol, 150 mM NaCl (total NaCl ~ 300 mM), 0.01% P20 detergent, 1 mM TCEP and 0.05% DMSO. For the binding of MGs pomalidomide and CC-220 to DDB1:*N*-avi-CRBN, the buffer contains 2% DMSO, and 1 mM EDTA. For the binding of SALL4 proteins to DDB1:*N*-avi-CRBN:MG complex, the Biacore ABA method was used to measure binding data for solutions of SALL4 zinc finger domains with 5 µM MG, which is constant throughout the acquisition of baseline, SALL4 association, and SALL4 dissociation. The temperature was 15 °C, and the flow rate was 30 µL/minute. In this experiment, only the solutions for the association phase contain a 2X dilution series of IKZF2 proteins. Thus, the ABA experiment measures the binding of SALL4 protein to a DDB1:CRBN:MG complex resulting in the formation of DDB1:CRBN:MG:SALL4 complex and the dissociation of SALL4 protein from this complex. Data analysis was performed with Biacore Insights software to normalize data relative to the baseline injections.

### Crystallization, data collection and structure determination

#### Crystallization of DDB1:CRBN in complex with SALL4 ZF1-2(379–432) and pomalidomide or CC-220

Pomalidomide or CC-220 (at 2 mM in DMSO) was added to 300 µL of CRBNΔ67:DDB1ΔBPB (177 µM) and to 226 µL SALL4 ZF1-2 (706 µM). After 20 min of incubation on ice, SALL4 ZF1-2/pomalidomide mixture or CC-220 was combined with the respective CRBNΔ67:DDB1ΔBPB/pomalidomide or CC-220 solution and further incubated on ice for 20 min. The complex was then concentrated to 354 µL (150 µM of complex) prior crystallization screening. The complexes were crystallized via the hanging drop vapor diffusion method (at 18 °C) using a 1 µl:1 µl drop ratio of protein and a well solution containing 0.2 M Sodium Malonate, 20% (w/v) PEG 3350. The crystals were harvested and cryoprotected using well solution with 2 mM ligand and 20% ethylene glycol. X-ray diffraction data was collected at the APS beamline ID17. The data were processed with autoPROC v1.1.7 (Global Phasing Ltd, United Kingdom). The initial structures were determined using molecular replacement (MR) via Phaser^17^ by CCP4^18^, with search models derived from PDB entry 6H0F for DDB1ΔBPB, and IKZF1 ZF1 separately. The structures were refined to convergence by iterative cycles of rebuilding with Coot^19^ and refinement with Phenix.refine^20^; ligand restraints were generated using Grade v1.2.20 (Global Phasing Ltd, United Kingdom). Data processing and refinement statistics are in Supplementary Data Table 1.

#### Crystallization of DDB1:CRBN with SALL4 ZF1-2(370–454) and pomalidomide

Pomalidomide at 2 mM was added to both 87 µL CRBNΔ67:DDB1ΔBPB (198 µM) and 100 mL SALL4 ZF1-2(370–454) (250 µM). After 20 min of incubation on ice, SALL4 ZF1-2(370–454)/pomalidomide mixture was combined with CRBNΔ67:DDB1ΔBPB/pomalidomide complex and further incubated on ice for 20 min. This complex was concentrated to 150 µM prior to crystallization screening. Crystals grew in the same conditions as SALL4 ZF1-2(379–432) with pomalidomide/CC-220, using hanging drop format, and were cryoprotected using well solution with 20% ethylene glycol. X-ray diffraction data was collected at the Advanced Light Source (ALS) 5.0.2 beamline. The data was processed with autoPROC (Global Phasing Ltd, United Kingdom). A molecular replacement solution was found with Phaser^17^ by CCP4, using the structure of DDB1:CRBN with SALL4 ZF1-2(379–432) and pomalidomide as a search model. The model was manually rebuilt and refined to convergence by iterative cycles of Coot^18^ and Phenix.refine^19^. Data processing and refinement statistics are in Supplementary Data Table 1.

### Structure visualizations and calculations

All structural visualizations, comparisons, molecular interactions and contact surface area calculations were generated or obtained using the molecular graphics program MOE (release 2022.02) by the Chemical Computing Group (Montreal, Canada).

### Supplementary Information

Supplementary Information.

## Data Availability

The data sets generated during and/or analyzed during the current study, including 8U15, 8U16, and 8U17 will be available at the time of publication at the RCSB Protein Data Bank, www.rcsb.org.
