# Supplementary material for: Structural and biophysical comparisons of the pomalidomide- and CC-220-induced interactions of SALL4 with cereblon
Source: Sci Rep. 2023 Dec 12;13:22088. doi: 10.1038/s41598-023-48606-3 (PMC10716131; doi:10.1038/s41598-023-48606-3)
Supplement: Supplementary file 1 — Supplementary Information. [file 41598_2023_48606_MOESM1_ESM.pdf]

Supplement

Table S1. X-ray crystallography statistics for 8U15, 8U16, and 8U17

Datasets	DBPB DDB1: D*N*-67CRBN:ZF1-2(379-432):CC-220 PDB ID: 8U15	DBPB DDB1: D*N*-67CRBN:ZF1-2(379-432):POM PDB ID: 8U16	DBPB DDB1: D*N*-67CRBN:ZF1-2(370-454):POM PDB ID: 8U17
**Diffraction-data statistics**			
X-ray source	APS ID17	APS ID17	ALS 5.0.2
Wavelength	1.00	1.00	1.00
Space group	P 2_1	P 2_1 2_1 2_1	P 2_1 2_1 2_1
Cell parameters			
a, b, c (Å)	110.73, 95.73, 150.95	95.54, 151.99, 218.46	116.08, 151.66, 195.38
α, β, γ (°)	90.00, 91.72, 90.00	90.00, 90.00, 90.00	90.00, 90.00, 90.00
Resolution range (Å)[a]	55.34-2.95 (3.06-2.95)	47.77-2.9 (3.00-2.9)	49.9-3.1 (3.21-3.10)
R-merge[b]	10.38 (93.71)	7.73 (92.16)	6.31 (212)
CC1/2	0.992 (0.38)	0.997 (0.55)	0.999 (0.47)
Mean I/σ (I)	5.22 (0.81)	5.18 (0.67)	7.73 (0.38)
Multiplicity	2.0 (2.0)	2.0 (2.0)	2.0 (2.0)
Completeness (%)	99.87 (99.86)	99.30 (99.14)	96.01 (66.86)
Total reflections	133313 (13266)	142439 (14016)	126489 (12488)
Unique reflections	66744 (6636)	71220 (7008)	63245 (6244)
**Refinement Statistics**			
R-work (%)	21.93 (35.17)	22.04 (40.16)	30.48 (49.80)
R-free (%)[c]	27.10 (38.77)	27.30 (42.63)	34.55 (52.84)
No. of non-hydrogen atoms	18548	18092	17033
Protein residues	2398	2410	2379
Solvent	13	36	0
Ligand	132	50	46
RMS deviation			
RMS (Å)	0.002	0.01	0.002
RMS (°)	0.52	1.15	0.52
Ramachandran plot			
Ramachandran favored (%)	93.85	94.2	89.67
Ramachandran allowed (%)	5.77	5.34	9.47
Ramachandran outliers (%)	0.38	0.46	0.86
Rotamer outliers (%)	3	4.57	0.25
Clashscore	7.8	10.62	5.12

[a] Value in parentheses is for the highest-resolution shell.
[b] Rmerge = $\Sigma_h \Sigma_i |I(h)_i - \langle I(h) \rangle| / \Sigma h \Sigma_i I(h)_i$, where I(h) is the intensity for reflection h, Σ_h is the sum for all reflections, and Σ_i is the sum for i measurements of reflection h.
[c] $R = \Sigma \,|\,|F_{obs}| - |F_{calc}|\,| / \Sigma\, |F_{obs}|$, where R_{free} is calculated for a randomly chosen 5% of reflections, which were not used for structure refinement, and R_{work} is calculated for the remaining reflections

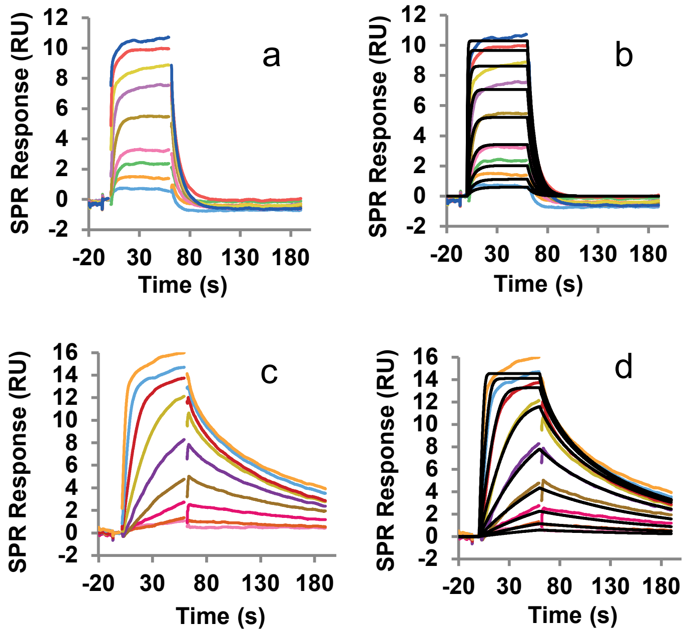

SPR Result	Pomalidomide (N=3)	CC-220 (N=3)
K_D (nM)	170±28	9±0.9
k_{on} (1/M*s)	$7.2 \times 10^5 \pm 1.1 \times 10^5$	$7.0 \times 10^6 \pm 2.1 \times 10^6$
k_{off} (1/s)	0.13±0.03	0.064±0.025
R_{max} (RU)	10.6±0.4	14.9±0.1

Figure S1: SPR data and 1:1 kinetic modeling of data for the interaction of pomalidomide (a, b, 9pt 2X dilution series, top concentration 2.5μM) and CC-220 (c, d, 9pt 2X dilution series, top concentration 0.25μM) with DDB1:CRBN.

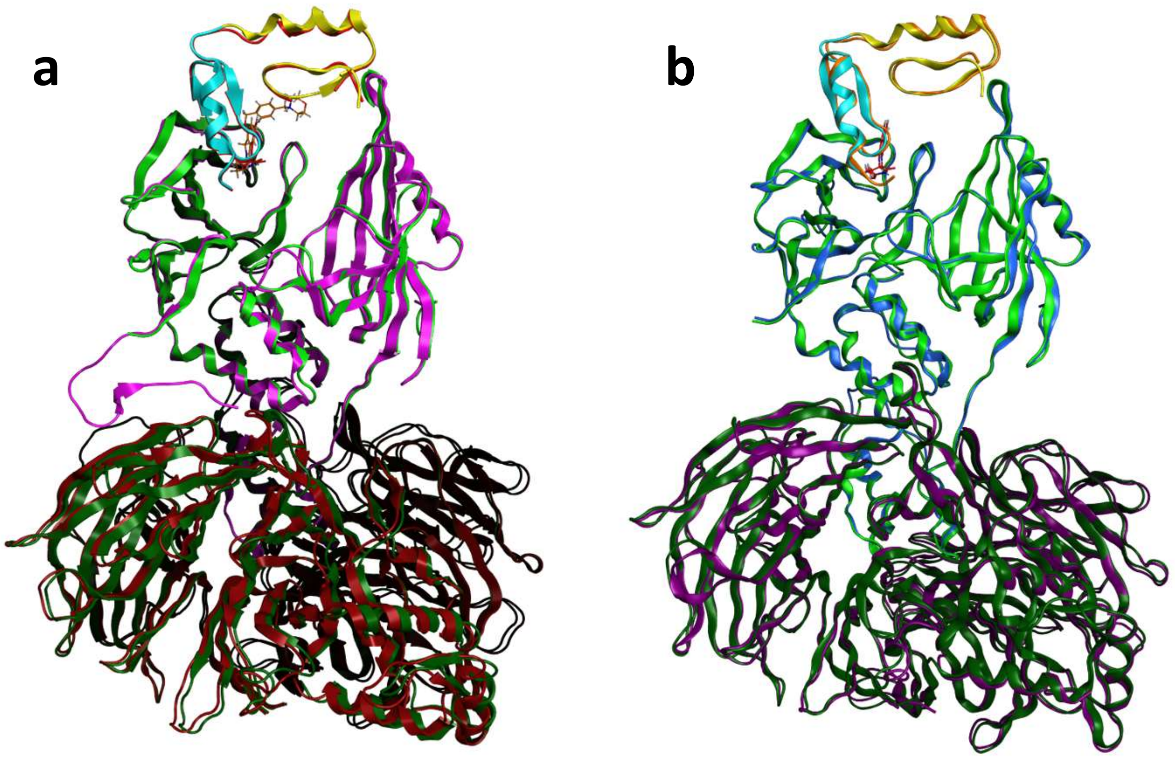

Figure S2. Structural overlays based on alignment of cereblon residues for **a)** DDB1:CRBN:POM:ZF1-2(379-432) and the corresponding CC-220/ZF1-2(379-432) structure. Color schemes: DDB1:CRBN:POM:ZF1-2(379-432) is dark green, green, and yellow/cyan, respectively and for the corresponding CC-220 structure, the corresponding colors are dark red, magenta, and red. **b)** DDB1:CRBN:POM:ZF1-2(379-432) and the corresponding POM/ZF1-2(370-454) structure. The ZF1-2(379-432) structure colors are dark green, green, and yellow/cyan and the ZF1-2(370-454) structure colors are purple, blue, and orange for DDB1, CRBN, and ZF1-2, respectively.

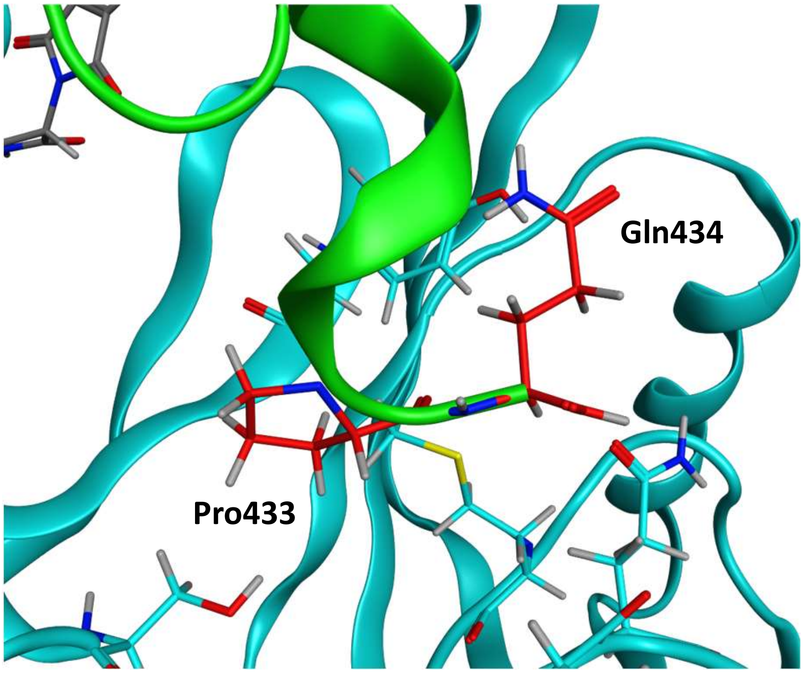

Figure S3: A model of the interactions of *C*-terminal SALL4 residues Pro433 and Gln434 in the DDB1:CRBN:POM:ZF1-2(370-434) structure.

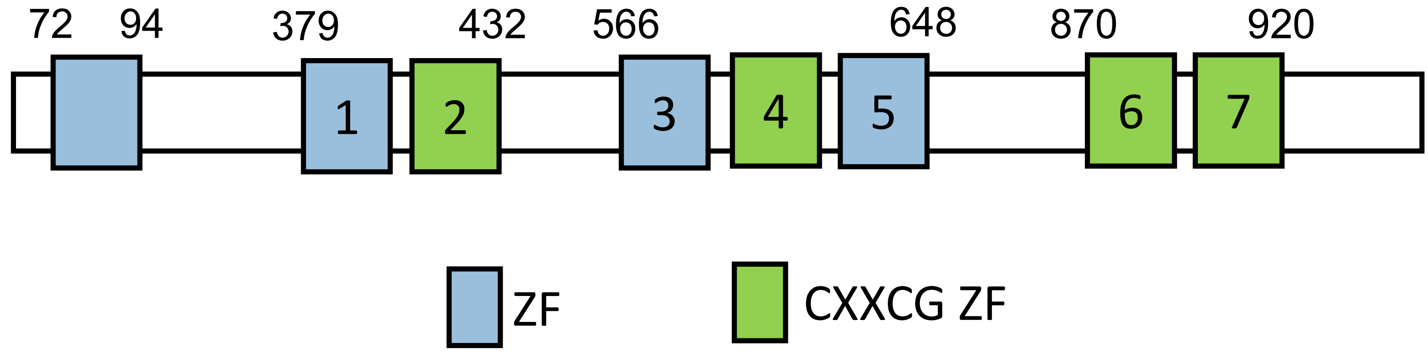

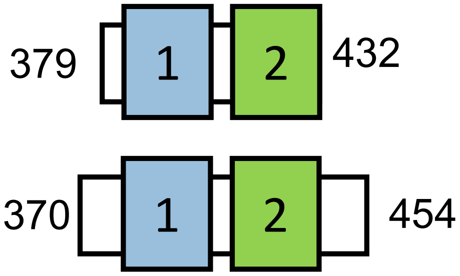

ZF1-2(379-432): SLYKHKCKYC SKVFGTDSSL QIHLRSHTGE RPFVCSVCGH RFTTKGNLKV HFHRH

ZF1-2(370-454): SDVKPKDEAA LYKHKCKYCS KVFGTDSSLQ IHLRSHTGER PFVCSVCGHR FTTKGNLKVHFHRHPQVKAN PQLFAEFQDK VAAGNG

Figure S4: Representation SALL4 zinc fingers and sequences of key zinc finger constructs. Designation of zinc finger 1 is based on prior literature studies. The ZF2 CXXCG motif that interacts with the CRBN:MG complex is underlined.
